# Moral sensitivity, spiritual care perception, and compassion fatigue of nurses caring for earthquake victims

**DOI:** 10.1111/inr.13066

**Published:** 2024-11-12

**Authors:** Turkan Karaca, Semiha Aydin Ozkan

**Affiliations:** ^1^ Faculty of Health Sciences, Nursing, Harran University‐Osmanbey Campus Haliliye/Şanlıurfa Sanliurfa Turkey; ^2^ Faculty of Health Sciences, Midwifery, Adiyaman University‐Altınşehir, Centre/Adiyaman Adiyaman Turkey

**Keywords:** Compassion fatigue, earthquake victims, moral sensitivity, spiritual care

## Abstract

**Background:**

Two major earthquakes occurred within 10 hours in Kahramanmaraş on February 6, 2023, which resulted in thousands of deaths and injured people within Turkey.

**Aim:**

To determine the relationship between moral sensitivity, spiritual care perception, and compassion fatigue among nurses caring for earthquake victims.

**Methods:**

The study population consisted of 483 nurses working in public, private, and university hospitals in earthquake‐affected areas in Turkey. The sociodemographic characteristics form, moral sensitivity questionnaire, spirituality and spiritual care rating scale, and compassion fatigue short scale were used for data collection.

**Results:**

There was a statistically significant negative relationship between nurses' moral sensitivity and compassion fatigue; in other words, as compassion fatigue increases, nurses' moral sensitivity decreases. A statistically significant negative relationship was found between nurses' perception of spiritual care and their compassion fatigue; in other words, as compassion fatigue increases, nurses' perception of spirituality and spiritual care decreases.

**Conclusion:**

This study provides valuable insights into moral sensitivity, perceptions of spiritual care, and compassion fatigue among nurses caring for earthquake victims.

**Implications for nursing and health policy:**

Minimizing nurses' compassion fatigue improves quality patient care, increases patient and employee satisfaction, and maintains commitment to the profession. To achieve this goal, it is essential to offer in‐service training, conferences, or seminar programs to nurses focused on fostering a sense of compassion. Nurses should be provided with environments that will improve their self‐care, and institutional policies and strategies should be developed to cope effectively with this situation.

## INTRODUCTION

Two major earthquakes occurred within nearly 10 hours in Kahramanmaraş in Turkiye on February 6, 2023 (Monday). The first, which occurred in the Pazarcık district, was of 7.8 magnitude, and the second was of 7.5 magnitude in the Elbistan district. These two severe earthquakes caused destruction in Kahramanmaraş, Hatay, Gaziantep, Osmaniye, Malatya, Adana, Diyarbakır, Şanlıurfa, Adıyaman, Kilis, and Elazığ cities in Turkey. Thus, the Disaster and Emergency Management Authority (AFAD, 2015) declared thousands of people dead (50 096) and injured (158 165) in Turkey. Nurses, who are important members of the healthcare team in natural disasters such as earthquakes, floods, and hurricanes, also continue their duties as rescue team members in hospitals. Therefore, it is crucial to assess the moral sensitivity, spiritual care perception, compassion fatigue, and physical needs of nurses caring for earthquake victims since the disaster occurred, as well as their physical needs.

## BACKGROUND

Compassion involves feelings of pity, spiritual care, kindness, sorrow, and interest in response to situations one encounters (Figley, 2002). It plays a significant role in the nursing profession, emphasizing the need for nurses to approach patients and their relatives with compassion (Mersin et al., 2020; Ondrejková & Halomava, 2022). Compassion fatigue refers to the state of mental and physical exhaustion that arises as a result of empathy and stress experienced in response to others' suffering or exposure to traumatic events (Marshman et al., 2022). This type of fatigue has been highlighted as a risk for individuals in caregiving professions (Cavanagh et al., 2020). Particularly, nurses tend to experience compassion fatigue while caring for patients whose lives are at risk (Storm & Chen, 2021). Compassion fatigue presents itself through physical (neglect of self‐care and health issues), emotional (excessive guilt, work‐related complaints, and nightmares), and social (isolation) symptoms, and can lead to legal issues (Gustafsson & Hemberg, 2022; Walker, 2020). These negative effects can adversely impact the quality of work life and performance quality of the employees, ultimately affecting institutions (Kartsonaki et al., 2023).

Research indicates that healthcare professionals may continue to experience symptoms of post‐traumatic stress disorder, depression, anxiety, and stress even after the crisis has ended (Altun et al., 2020). Compassion fatigue has been shown to adversely impact both patients and healthcare professionals, resulting in higher resignation rates, reduced performance, strained colleague relationships, decreased life satisfaction, and increased care costs due to inadequate quality of care services.

Nurses experiencing compassion fatigue may struggle to recognize or comprehend their patients' conditions, leading to the neglect of interventions that support patient autonomy due to burnout and desensitization (Powell, 2020). Consequently, nurses may display indifference, reluctance, and anger toward the patient.

An increase in compassion fatigue may cause increased complaints from patients and their relatives, communication problems, and decreased moral sensitivity in nurses (Tanrıkulu & Ceylan, 2021). Moral sensitivity can be defined as the ability to recognize ethical issues and make decisions quickly and accurately. An increase in compassion fatigue and a decrease in moral sensitivity in nurses can lead to serious outcomes in patient care/treatment, such as complications, morbidity, and even mortality (Aslan et al, 2022). As nurses care for patients who are both physically and psychologically suffering, such as earthquake victims, compassion fatigue is frequently observed in the nursing profession (Powell, 2020). Previous studies indicate that nurses, especially those continuously and extensively exposed to others' traumas, are at high risk for compassion fatigue and moral issues (Xie et al., 2021).

Nurses working in areas like palliative care or intensive care, where patients, such as disaster victims, are unlikely to return to their previous health or well‐being, are also at heightened risk for developing compassion fatigue. Nurses encounter numerous physical and mental events that can adversely affect their health or the quality of patient care, resulting in compassion fatigue and they need spiritual care support. Spiritual care, however, refers to the support and emotional response of healthcare professionals toward individuals who have experienced traumatic events (Gustafsson & Hemberg, 2022), but unlike compassion, it is sometimes considered a negative attitude (Eisenberg & Stayer, 1990; Özcan, 2020). The feeling of compassion with spiritual care is closely linked to kindness and the instinct to protect others (Kırçı & Kızıler, 2021; Nas & Sak, 2020). Spiritual care is a personal practice rooted in spiritual experiences that support individuals through illness and aid in improving their overall well‐being. It involves the desire to find the meaning of life, hope, forgiveness, and love, and the need for speech rituals, worship, and prayer (Willemse et al., 2020).

In the literature, some of the initial symptoms of compassion fatigue observed in nurses include insensitivity to spiritual needs, feelings of hopelessness, and a loss of meaning (Kutlu et al., 2020; Xie et al., 2021).

From this perspective, due to the lack of existing research, our study questions are as follows:

Is there a significant difference between the moral sensitivity, spirituality, and spiritual care and compassion fatigue mean scores and the independent variables?

What are nurses’ moral sensitivity, spirituality, and spiritual care and compassion fatigue mean scores?

What is the relationship between moral sensitivity, spiritual care perception, and compassion fatigue among nurses caring for earthquake victims?

This study aimed to determine the moral sensitivity, spiritual care perception, and compassion fatigue of nurses working in the region following the earthquake in Turkiye on February 6, 2023, and the relationship between these concepts.

## METHODS

### Design

This descriptive‐correlational study was conducted to determine the moral sensitivity, spiritual care perception, and compassion fatigue of nurses caring for earthquake victims and the relationship between these concepts.

### Participants and setting

The study population included the nurses working in public, private, and university hospitals’ palliative care and intensive care units in earthquake zones (10 cities) in Turkey. In response to the significant number of serious injuries caused by the earthquakes, health authorities made the critical decision to repurpose all sui hospital wards, excluding emergency services, into intensive care and palliative care units. The number of nurses to be included in the sample was calculated using the A‐priori Sample Size Calculator for Multiple Regression website, and a minimum of 472 participants were reached an alpha level of 0.05, the effect size of 0.15 and 0.95 power. The study sample consisted of 483 nurses who cared for earthquake victims and volunteered to participate.

### Ethical approval

The ethics committee approval and the written permission for the study were obtained from the Scientific Research Ethics Committee of the University (number no: 341) and the Ministry of Health in Turkiye, respectively. Data were collected using a form created in Google Forms, with an informed consent statement included on the first page. Only participants who read and approved the informed consent statement were included in the study, ensuring that their consent was obtained. Additionally, the necessary permissions were obtained from the authors to use the measurement tools in the study.

### Data collection

Between April and September 2023, data were collected using an online form created by the researchers and shared with nurses working in palliative and intensive care units across public, private, and university hospitals in the earthquake‐affected areas (10 cities) of Turkey via social media platforms. On the first page of the online data collection form, participants were asked to provide their informed consent by filling out an informed consent form that outlined the study's purpose, duration, content, data storage policies, and the researchers' contact information.

The next page of the data collection form, containing the questions, was accessible only to participants who had given their consent. Those who chose not to consent were exited from the study. On average, participants took approximately 12 to 15 minutes to complete the form.

### Data collection tools

The Sociodemographic Characteristics Form includes questions prepared by the researchers to gather information about the nurses' sociodemographic characteristics, including age, gender, marital status, and years of experience. Age was categorized into three groups: 21–29, 30–39, and 40–48. This classification aligns with similar groupings found in the literature, allowing the study sample to be organized within these age ranges (Arslan et al., 2017; Gustafsson & Hemberg, 2022; Tazegün & Çelebioğlu, 2016).

Moral sensitivity was measured using the Turkish version of the Moral Sensitivity Questionnaire (MSQ) (Tosun, 2018). Developed by Kim Lutzen to assess moral sensitivity, it was first used in psychiatric clinics at Karolinska Nursing Institute in Stockholm (Sweden) in 1997. It was later used in other clinics with physicians and nurses in the same institution. This self‐administered questionnaire is structured as a seven‐point Likert scale and includes 27 statements representing six subdimensions for scoring. The test is divided into six categories: autonomy (professional responsibility), reliance on medical authority (patient‐oriented care), moral meaning, expressing benevolence, experiencing conflict, and interpersonal orientation. Three items (items 3, 23, and 26) are not included in any subdimensions (Lutzen et al., 1997; Tosun, 2018). The responses for each statement range from “I totally disagree” to “I totally agree.” A score of 1 to 7 for each item reflects the degree of agreement or disagreement with the statement. Higher scores indicate lower moral sensitivity, while lower scores suggest greater moral sensitivity. The Turkish version of the questionnaire was validated and found reliable by Tosun in 2003, with a Cronbach's alpha of 0.82. In the present study, the Cronbach's alpha was calculated to be 0.78.

Spirituality and Spiritual Care Rating Scale (SSCRS) was developed by McSherry. The SSCRS measures spirituality by quantifying participants’ perceptions of the extent to which they hold certain spiritual views and engage in certain spiritually related activities. It is a Likert‐type scale, consisting of a total of 17 items and 3 subdimensions, answered on a five‐point scale from “strongly disagree (1 point)” to “strongly agree (5 points).” These subdimensions are spirituality and spiritual care (6, 7, 8, 9, 11, 12, and 14), individual care (1, 2, 10, and 15), and religiosity (4, 5, 13, and 16). Four (3, 4, 13, and 16) items on the scale are reverse scored.

The possible scores range from 17 to 85. In general, higher scores indicate a higher level of perception of spirituality or the provision of spiritual care. Ergül and Bayık (2007) conducted a study to assess the validity and reliability of the Turkish version of the scale. The Turkish version of SSCRS demonstrates satisfactory validity and reliability (Ergül & Bayık, 2007). The Cronbach's alpha value reported for this study was 0.82.

The Compassion Fatigue Short Scale (CFSS), developed in 2006, was translated into Turkish and tested for validity and reliability by Dinç and Ekinci. It consists of 13 items and 2 subdimensions. Items “3, 5, 8, 10, and 12” in the scale represent the secondary trauma subdimension; “1, 2, 4, 6, 7, 9, 11 and 13” items measure the occupational burnout subdimension. The scale uses a 10‐point Likert system, where responses range from 1 (rarely/never) to 10 (very often) for each statement. Scores for individual items vary between 1 and 10. After marking the 13 items, the total score obtained determines the level of compassion fatigue indicated. The lowest score on the scale is 13 and the highest is 130. As the score increases, the level of compassion fatigue experienced by the individual also increases. In the reliability study conducted by Dinç and Ekinci in 2019, the overall Cronbach's alpha coefficient for the CFSS was found to be 0.87. When examining the reliability coefficients, the Cronbach's alpha value for the secondary trauma subscale was found to be 0.74, while the Cronbach's alpha value for the professional burnout subscale was 0.85 (Dinç & Ekinci, 2019). The overall Cronbach's alpha value for this study was found to be 0.76.

### Data analysis

Data analysis was carried out with the data of 483 participants using the IBM SPSS Statistics 26 package program. Before analyzing the data, the Kolmogorov–Smirnov and Shapiro–Wilk tests were applied to determine whether the dependent variables were normally distributed. According to the test result, as *p* was < 0.005, the data were analyzed with nonparametric tests. In the analysis of the study data, categorical variables were shown with a number and percentage while the mean, standard deviation, and median for the numerical variables. The Mann–Whitney *U* test and the Kruskal–Wallis test were used to test the differences between the groups. Among the post hoc tests, LSD and Tukey tests were used to identify which specific groups showed significant differences for variables that were significant in groups of three. Interpretation of the relationships between two independent numerical variables was performed with the Spearman correlation coefficient. Simple linear regression analysis was used to determine how much moral sensitivity and spiritual care scores of the nurses affected compassion fatigue. Statistical significance was interpreted at the level of 0.05 in the analyses.

## RESULTS

Although the mean age of the participants was 31.67 ± 7.22 (min 21–max 48) years, 45.3% were in the 21–29 age group, 58.2% were women, and 66.3% were married. Most of the participants (50.7%) were working in internal units. Although 38.9% of them had been working in the same institution for 11 years or more, the mean working year of the participants was 9.52 ± 6.37 (min 1–max 25).

When they were examined in terms of age and duration of working years, the mean MSQ score of nurses who were between the ages of 30–39 (84.19 ± 6.10) and who had been working for 6–10 years (85.22 ± 4.47) was determined to be statistically significantly higher than the other groups (*p* < 0.05). When the SSCRS scores of the participants were examined according to sociodemographic variables, they showed a similar distribution in terms of gender, marital status, and the unit they worked in (*p* > 0.05).

However, when the participants were examined in terms of age and duration of working years, the mean scores of SSCRS in nurses who were between 21 and 29 years old (50.03 ± 2.60) and who had worked between 1 and 5 years (50.35 ± 2.65) were found to be statistically significantly higher than the other groups (*p* < 0.05).

However, when the mean CFSS score of the participants was examined according to sociodemographic variables, they had a similar distribution in terms of age, gender, marital status, and the unit they worked in (*p* > 0.05). However, when analyzed based on years of experience, the mean CFSS score for nurses with 11 or more years of service (88.42 ± 2.39) was found to be statistically significantly higher than for those with 1–5 years (87.11 ± 2.88) and 6–10 years (87.52 ± 3.07) of experience (*p* < 0.05). When the participants' mean MSQ scores were examined according to sociodemographic variables, they were determined to show a similar distribution in terms of gender, marital status, and the unit they worked in (*p* > 0.05) (Table 1).

**TABLE 1:** Distribution of mean scores of MSQ, SSCRS, and CFSS according to sociodemographic characteristics (*n* = 483).

Sociodemographic characteristics		MSQ	SSCRS	CFSS
	*n* (%)	Mean ± SD	Mean ± SD	Mean ± SD
Age				
21–29	219 (45.3)	82.42 ± 6.10	50.03 ± 2.60^*^	87.88 ± 2.77
30–39	182 (37.7)	84.19 ± 6.10^*^	48.90 ± 2.40	88.04 ± 2.82
40–48	82 (17.0)	82.16 ± 5.17	49.37 ± 3.39	88.70 ± 2.49
Statistics		KW = 13.077 *p* = 0.001	KW = 16.719 *p* = 0.000	KW = 5.552 *p* = 0.062
Gender				
Male	202 (41.8)	83.34 ± 6.13	49.42 ± 2.63	88.14 ± 3.14
Female	281 (58.2)	82.83 ± 5.93	49.54 ± 2.79	88.04 ± 2.45
Statistics		Z(U) = −0.608 *p *= 0.543	Z(*U*) = −0.505 *p* = 0.614	Z(U) = −1.078 *p* = 0.281
Marital status				
Married	320 (66.3)	83.03 ± 6.07	49.36 ± 2.76	88.26 ± 7.74
Not married	163 (33.7)	83.07 ± 5.91	49.77 ± 2.63	87.73 ± 2.76
Statistics		Z(U) = −0.466 *p* = 0.641	Z(U) = −1.283 *p* = 0.199	Z(U) = −1.764 *p* = 0.078
Working year				
1–5 years	170 (35.2)	81.89 ± 6.30	50.35 ± 2.65^*^	87.11 ± 2.88
6–10 years	125 (25.9)	85.22 ± 4.47^*^	48.93 ± 2.09	87.52 ± 3.07
11 years or more	188 (38.9)	82.63 ± 5.74	49.09 ± 2.97	88.42 ± 2.39^*^
Statistics		KW = 14.388 *p* = 0.001	KW = 24.098 *p* = 0.000	KW = 11.422 *p *= 0.003
Working unit				
Internal units	245 (50.7)	83.26 ± 5.43	49.31 ± 2.82	88.26 ± 2.59
Surgery units	238 (49.3)	82.81 ± 6.56	49.68 ± 2.61	87.90 ± 2.91
Statistics		Z(U) = −0.797 *p* = 0.426	Z(U) = −1.730 *p* = 0.084	Z(U) = −1.747 *p* = 0.081

Z(U) = Mann–Whitney *U* test, KW** = **Kruskal–Wallis test

.* The group that created the significant difference was identified using the LSD and Tukey tests.

The MSQ mean score of the participants was 83.05 ± 6.02. Modifying autonomy, reliance on medical authority, moral meaning, expressing benevolence, experiencing conflict, experiencing conflict, interpersonal orientation subdimension scores were 22.59 ± 1.63, 13.78 ± 1.88, 13.03 ± 1.95, 6.99 ± 1.79, 12.32 ± 2.28, and 14.33 ± 2.07, respectively. The SSCRS score was 49.49 ± 2.73. Spirituality and spiritual care, individual care, and religiosity subdimension scores were 24.03 ± 2.65, 15.92 ± 0.99, and 9.54 ± 0.81 respectively. The CFSS score of the participants was 88.08 ± 2.76, the secondary trauma score was 34.71 ± 3.28, and the professional burnout score was 53.37 ± 3.37 (Table 2).

**TABLE 2:** Nurses' MSQ, SSCRS, and CFSS total scale and subscales (*n* = 483).

Scales	Minimum	Maximum	Median	Mean ± SD
MSQ	65.00	99.00	85.00	83.05 ± 6.02
Modifying autonomy score (professional responsibility)	18.00	27.00	23.00	22.59 ± 1.63
Reliance on medical authority (patient‐oriented care)	10.00	18.00	13.00	13.78 ± 1.88
Moral meaning	8.00	17.00	13.00	13.03 ± 1.95
Expressing benevolence	4.00	10.00	7.00	6.99 ± 1.79
Experiencing conflict	8.00	17.00	12.00	12.32 ± 2.28
Interpersonal orientation	10.00	17.00	15.00	14.33 ± 2.07
SSCRS	44.00	56.00	50.00	49.49 ± 2.73
Spirituality and spiritual care	18.00	28.00	25.00	24.03 ± 2.65
Individual care	14.00	19.00	16.00	15.92 ± 0.99
Religiosity	3.00	11.00	10.00	9.54 ± 0.81
CFSS	81.00	97.00	89.00	88.08 ± 2.76
Secondary trauma	28.00	40.00	36.00	34.71 ± 3.28
Professional burnout	47.00	60.00	54.00	53.37 ± 3.37

There was a a statistically significant negative relationship between nurses' moral sensitivity and their perception of spiritual care, in other words, as moral sensitivity increased, nurses' perception of spiritual care decreased (*r* = −0.552, *p* < 0.001). The relationship between nurses' moral sensitivity and their compression fatigue was statistically significant and negative, in other words, as moral sensitivity increased, nurses' compression fatigue decreased(*r* = −305, *p* < 0.001) (Table 3).

**TABLE 3:** Relationships between MSQ, SSCRS, and CFSS (*n* = 483).

Measurements	*MSQ*	Modifying autonomy score (professional responsibility)	Expressing benevolence	Moral meaning	Experiencing conflict	Reliance on medical authority (patient‐oriented care)	Interpersonal orientation
**SSRCS**							
*r*	−0.552^**^	−0.137^**^	−0.188^**^	0.153^**^	−0.089	−0.560^**^	−0.658^**^
*p*	0.000	0.003	0.000	0.001	0.051	0.000	0.000
Spirituality and spiritual care							
*r*	−0.527^**^	−0.063	−0.304^**^	0.191^**^	0.097^*^	−0.618^**^	−0.647^**^
*p*	0.000	0.169	0.000	0.000	0.033	0.000	0.000
Individual care							
*r*	−0.022	−0.162^**^	0.066	0.338^**^	−0.412^**^	0.041	−0.341^**^
*p*	0.633	0.000	0.148	0.000	0.000	0.369	0.000
Religiosity							
*r*	0.043	−0.041	0.248^**^	−0.302^**^	−0.270^**^	0.340^**^	0.265^**^
*p*	0.343	0.371	0.000	0.000	0.000	0.000	0.000
**CFSS**							
*r*	−0.305^**^	−0.268^**^	−0.605^**^	0.020	0.395^**^	−0.109^*^	−0.277^**^
*p*	0.000	0.000	0.000	0.657	0.000	0.016	0.000
Secondary trauma							
*r*	−0.406^**^	−0.179^**^	−0.037	−0.504^**^	−0.043	−0.030	−0.067
*p*	0.000	0.000	0.413	0.000	0.343	0.506	0.144
Professional burnout							
*r*	0.209^**^	0.098^*^	−0.338^**^	0.520^**^	0.418^**^	−0.128^**^	−0.093^*^
*p*	0.000	0.031	0.000	0.000	0.000	0.005	0.040

*r*: Spearman correlation coefficient.

* *p* < 0.05.

** *p* < 0.01.

In Table 4, which presents the findings of the simple regression analysis, it was found that nurses' MSQ scores had an effect of 0.078 on CFSS and an effect of 0.253 on SSCRS scores. In other words, a one‐unit increase in the MSQ score resulted in a 25.3% decrease in spirituality and spiritual care and a 7.8% decrease in compassion fatigue (Table 4).

**TABLE 4:** Effect of CFSS and SSCRS on nurses' MSQ with simple linear regression analysis.

Scales	Unstandardized coefficients		Standardized coefficients		
	*B*	Standardized error	Beta	*t*	*P*
(Constant) SCFF	136.574 −0.608	8.420 0.096	−0.279	16.219 −6.360	0.000
	*R* = 0.279	*R* ^2 ^= 0.078	*F *= 40.450	*p* = 0.000	
(Constant) SSCRS	137.989 −1.110	4.311 0.087	−0.503	32.011 5.310	0.000
	*R* = 0.503	*R* ^2 ^= 0.253	*F* = 162.952	*p* = 0.000	

Dependent variable: MSQ.

## DISCUSSION

In our study, MSQ scores of nurses differ significantly according to their age groups and duration of working years. Similar to the results of this study, Kılıç et al. (2020) and Tazegün and Çelebioğlu (2016) found a significant difference between age and MSQ in their studies. However, when the moral sensitivities of nurses were examined according to their ages in the study, the moral sensitivities of the 30–39 age group were found to be higher. This may be because nurses with greater professional experience tend to develop a heightened sensitivity to moral issues as their careers progress. Similar to our study, there are studies (Arslan & Calpbinici, 2018; Temiz et al., 2017) that reported a significant relationship between the level of moral sensitivity according to the year of study. The moral sensitivity of nurses with 1–14 years of experience was noted to be higher than that of nurses with 15–29 years of experience in Yorulmaz (2021).

There was a statistically significant difference between the SSCRS mean scores of the participants according to age and duration of working years. Contrary to our findings, Tambağ et al. (2018) and Çelik et al. (2024) reported that the variability in their study duration throughout the day and year did not impact spirituality and spiritual care. The differences between these studies and our own may be due to the variations in the individuals studied and the specific characteristics of those participants.

In our study, nurses' CFSS scores differ significantly according to their duration of working years. Also, it was found that compassion fatigue increased as nurses' working years increased.

Some studies found that the duration of working years is a significant variable for compassion fatigue (Aslan & Yağcı Özen, 2021; Korkmaz et al., 2022), while other studies have found it not to be a significant variable (Çelebi & Ozturk, 2022; Tanrikulu & Ceylan, 2021). Similarly, Koca (2018) highlighted that nurses' compassion fatigue levels showed a statistically significant increase as the duration of working years in the profession increased. This result may have been influenced by the combination of prolonged working years and the added burden of severe disaster conditions experienced by nurses.

The MSQ mean score of the participants in our study was 83.05 ± 6.02. The average moral sensitivity identified in this study was lower than the mean scores reported in other studies in the literature. In other words, the findings indicate that nurses in this study showed higher levels of moral sensitivity compared with those in previous research. The mean moral sensitivity in studies that reported lower levels than the findings of this study was 100.70 ± 27.47 (Uruf, 2022), 94.13 ± 25.88 (Özkan, 2021), 107.37 ± 34.72 (Oğuzhan et al., 2019), 93.06 ± 18.49 (Yılmaz, et al., 2018), 100.11 ± 21.15 (Fırat et al., 2017), and 124.20 ± 31.40 (Karaca, 2018). The reasons for the higher level of moral sensitivity observed in this study compared with the literature is that the research was conducted with nurses caring for earthquake victims. The individuals to whom nurses provide care are individuals in disaster situations, different from many other nursing fields.

The SSCRS mean score was 49.49 ± 2.73. The high item scores or the total score obtained from the scale indicate that the participants have a clear understanding of spirituality and spiritual care, reflecting a strong perception of these concepts. The results of the studies examining nurses' perceptions and ideas about spirituality and spiritual care in the literature are also similar to ours (Irmak, 2018; Kalkım et al., 2019; Kavak et al., 2014). The findings highlighted the additional characteristics of participants in their ability to provide spirituality and spiritual care.

The CFSS mean score of the participants in this study was 88.08 ± 2.76. A low score on the scale indicates low compassion fatigue and a high score indicates high fatigue. Nurses experienced more than half a point on the compassion fatigue scale. The mean compassion fatigue score identified in this study is higher than the mean scores reported in the literature. The mean scores of studies in the literature were 80.78 ± 16.93 (Özhan, 2022), 72.34 ± 22.15 (Karakurt et al., 2022), and 70.12 ± 28.94 (Özgünay et al., 2022). As nurses work with earthquake victims, who require increased empathy, compassion, and understanding, nurses working in disaster conditions may be more inclined to display these qualities. This heightened tendency to show compassion and empathy could contribute to an increase in compassion fatigue. Our study revealed that as moral sensitivity increases, nurses' perception of spirituality and spiritual care and compression fatigue decreases. Similar to our study, research in the literature has identified a relationship between these scales (Ateş, 2023; Ünlügedik & Akbaş, 2023; Vafaenina et al., 2024). In their study with 167 nurses working in the palliative care units of hospitals in Turkey, Ünlügedik and Akbaş (2023) reported a statistically significant negative relationship between moral sensitivity and their perception of spiritual care.

Furthermore, a statistically significant negative relationship was noted between moral sensitivity and compassion fatigue in Ateş (2023) with nurses working in a health institution in Istanbul. Vafaenina et al. (2024) reported a significant direct relationship between moral sensitivity and compassion fatigue. These results indicate that the challenges faced by nurses caring for earthquake victims significantly affect their moral sensitivities, spiritual care perceptions, and compassion fatigue. The negative relationship between moral sensitivity and compassion fatigue, in particular, suggests that these challenging conditions could adversely affect nurses' moral decision‐making processes.

Various professional groups have been the subject of study in the literature for nearly a decade. However, nursing, among all healthcare services professionals, is known to be more susceptible to burnout, compassion fatigue, and reduced compassion satisfaction due to physical and psychological stress associated with extensive patient contact. It is known that the increasing compassion fatigue and decreasing compassion satisfaction adversely affect the mental and physical health of nurses, ultimately leading to a decline in the quality of services they provide.

### Limitations of the study

This study has some limitations. First, the study was cross‐sectional. Second, the study was conducted with nurses who were working in public, private, and university hospitals in earthquake zones and caring for the earthquake victims, and its results are limited to this sample group and cannot be generalized.

## CONCLUSİON

This study provides valuable insights and findings regarding compassion fatigue, moral sensitivity, and the perception of spiritual care among nurses caring for earthquake victims. One of the key findings is that there is a statistically significant negative relationship between nurses' compassion fatigue and moral sensitivity; in other words, as compassion fatigue increases, nurses' moral sensitivity decreases. Second, nurses' compassion fatigue and their perception of spiritual care have a statistically significant negative relationship; in other words, as compassion fatigue increases, nurses' perception of spirituality and spiritual care decreases. There is a need for further studies to investigate, discuss, and evaluate compassion fatigue in primary healthcare practices to help inform best care initiatives, and nurses' actions towards patients and contribute to patient satisfaction.

### Implications for nursing and health policy

The concept of compassion plays a vital role in the relationship between patients and healthcare providers, as it is closely linked to the quality of care provided. Minimizing nurses' compassion fatigue improves quality patient care, increases patient and employee satisfaction, and maintains commitment to the profession. To achieve this goal, it is essential to offer in‐service training, conferences, or seminar programs focused on the concept of compassion for nurses. Additionally, nurses should be given opportunities to enhance their self‐care, and institutional policies and strategies should be developed to effectively cope with this situation.

## ETHICAL CONSIDERATION

This study was approved by the University of Ethical Board (Permission no: 341, March 3, 2023). Participants completed the questionnaire after agreeing to complete the survey through the link provided.

## AUTHOR CONTRIBUTIONS

Study concept and design: Türkan KARACA and Semiha AYDIN ÖZKAN. Collection of data: Türkan KARACA. Analysis and interpretation of data: Semiha AYDIN ÖZKAN. Drafting of the manuscript: Türkan KARACA and Semiha AYDIN ÖZKAN. Critical revision of the manuscript for important intellectual content: Türkan KARACA. Study supervision: Türkan KARACA and Semiha AYDIN ÖZKAN.

## CONFLICT OF INTEREST STATEMENT

The authors declared no potential conflicts of interest with respect to the research, authorship, and/or publication of this article.

## FUNDING INFORMATION

This research did not receive any specific grant from funding agencies in the public, commercial, or not‐for‐profit sectors.

## References

[inr13066-bib-0001] AFAD (2015) Basic legislation regarding disasters and emergency situations. Ankara. Available at: https://edirne.meb.gov.tr/meb_iys_dosyalar/2015_03/31040322_afet_ve_acil_durumlara_iliskin_temel_mevzuat.pdf [Accessed 29th May 2023].

[inr13066-bib-0002] Altun, Ö. , Kabakçı, K. & Olçun, Z. (2020) Self‐compassion in nursing. Journal of Archive Literature Review, 29(3), 218–225. 10.17827/aktd.593594

[inr13066-bib-0003] Arslan, F.T. & Calpbinici, P. (2018) Moral sensitivity, moral experiences and related factors of pediatric nurses: a cross‐sectional, correlational study. Acta Bioethica, 24(1), 9–18.

[inr13066-bib-0004] Aslan, H. , Erci, B. & Pekince, H. (2022) Relationship between compassion fatigue in nurses, and work‐related stress and the meaning of life. Journal of Religion and Health, 61(3), 1848–1860. 10.1007/s10943-020-01142-0 33386572 PMC7775832

[inr13066-bib-0005] Aslan, Ş. & Yağcı, Ö.M. (2021) Emotional ıntelligence and compassion fatigue in health workers in the context of socio‐demographic variables. Ekev Akademy Journal, 85, 435–452.

[inr13066-bib-0006] Ateş, A.Y. (2023) The relationship between the level of compassion and ethical sensitivity in nurses (an example of a health institution). Balkan Health Science Journal, 2(2), 41–52.

[inr13066-bib-0007] Cavanagh, N. , Cockett, G. , Heinrich, C. , Doig, L. , Fiest, K. , Guichon, J.R. & Doig, C.J. (2020) Compassion fatigue in healthcare providers: a systematic review and meta‐analysis. Nursing Ethics, 27(3), 639–665. 10.1177/0969733019889400 31829113

[inr13066-bib-0008] Çelebi, E. & Öztürk, C.H. (2022) Compassion satisfaction, burnout and compassion fatigue within the context of the dimensions of the professional quality of life scale in nurses: a cross‐sectional study. Turkiye Klinikleri Journal of Nursing Sciences, 14(1), 1–10. 10.5336/nurses.2021-82609

[inr13066-bib-0009] Çelik, Y. & Sivrikaya, S.K. (2024) Spiritual care approach of nurses working at internal clinics. Kocatepe Medical Journal, 25(1), 1–7.

[inr13066-bib-0010] Dinç, S. & Ekinci, M. (2019) Turkish adaptation, validity and reliability of compassion fatigue short scale. Current Approaches in Psychiatry, 11, 192–202. 10.18863/pgy.590616

[inr13066-bib-0011] Eisenberg, N. & Strayer, J. (1990) Empathy and its development. USA, Cup Archive.

[inr13066-bib-0012] Ergül, Ş. & Bayık, A. (2007) Validity and reliability of the spirituality and spiritual care rating scale Turkish version. Journal of Ege University Nursing School, 23(1), 75–87.

[inr13066-bib-0013] Figley, C. (2002) Compassion fatigue: psychotherapists chronic lack of self‐care. Journal of Clinical Psychology, 58(11), 1433–1441. 10.1002/jclp.10090 12412153

[inr13066-bib-0014] Fırat, B. , Karataş, G. , Barut, A. , Metin, G. & ve Sarı, D. (2017) Investigation of ethical sensitivities of emergency service nurses. E‐journal of Dokuz Eylül University Nursing Faculty, 10(4), 229–235.

[inr13066-bib-0015] Gustafsson, T. & Hemberg, J. (2022) Compassion fatigue as bruises in the soul: a qualitative study on nurses. Nursing Ethics, 29(1), 157–170. 10.1177/09697330211003215 34282669 PMC8866753

[inr13066-bib-0016] Irmak, H. (2018) The relationship between spiritual care practices, perceptions and competencies of psychiatric nurses, (Master's thesis). Manisa: Celal Bayar University Institute of Health Sciences.

[inr13066-bib-0017] Kalkım, A. , Dağhan, Ş. & Midilli, T.S. (2019) Nursing academicians’ perceptions of spirituality and spiritual care and their competence in spiritual care. Journal of Health Sciences and Professions, 6(2), 380–389. 10.17681/hsp.454678

[inr13066-bib-0018] Karaca, T. (2018) Examining the moral sensitivity of nursing students. Journal of Health Sciences and Professions, 5(1), 24–30. 10.17681/hsp.319379

[inr13066-bib-0019] Karakurt, P. , Fırat, M. & ve Yıldırım, S. (2022) Determining conscience perception and compassion fatigue among the nurses who worked at pandemic clinics: sample of city hospital. Gevher Nesıbe Journal of Medical and Health Sciences, 7(16), 60–68. 10.46648/gnj.309

[inr13066-bib-0020] Kartsonaki, M.G. , Georgopoulos, D. , Kondili, E. , Nieri, A.S. , Alevizaki, A. , Nyktari, V. & Papaioannou, A. (2023) Prevalence and factors associated with compassion fatigue, compassion satisfaction, burnout in health professionals. Nursing in Critical Care, 28(2), 225–235. 10.1111/nicc.12769 35315181

[inr13066-bib-0021] Kavak, F. , Mankan, T. , Polat, H. , Çıtlık, S.S & Sarıtaş, S. (2014) Nurses comments on the maintenance of spiritual care. Annals of Health Sciences Research, 3, 21–24.

[inr13066-bib-0022] Kılıç, D. , Bakan, A.B. , Aslan, G. & Uçar, F. (2020) Personal hygiene material usage levels of vocational technical training center students. Journal of Adnan Menderes University Health Sciences Faculty, 4(1), 20–29.

[inr13066-bib-0023] Kırçı, T. & Kızıler, E. (2021) The invisible side of the iceberg: compassion fatigue in nurses. Turkish Journal of Health Sciences and Research, 4(3), 11–21.

[inr13066-bib-0024] Koca, F. (2018) Compassion fatigue in nurses and investigation of effective factors, (master's thesis). Istanbul: Maltepe University, Institute of Health Sciences, Department of Surgical Nursing.

[inr13066-bib-0025] Korkmaz, M. , Avcı, İ.A. , Kırmacı, N.D. , Korkmaz, D. & Soyanıt, Ş. (2022) Evaluation of cultural competences and factors affecting cultural competences of nurses working in a university hospital. Gümüşhane University Journal of Health Sciences, 11(4), 1474–1483. 10.37989/gumussagbil.1094276

[inr13066-bib-0026] Kutlu, Ö. , Ermin, C. & Aygin, D. (2020) The relationshıp evaluation of intensive care nurses spiritual well‐being and spiritual care perceptions. Sakarya University Journal of Holistic Health, 3(3), 130–142.

[inr13066-bib-0027] Lutzen, K. , Evertzon, M. & Nordin, C. (1997) Moral sensitivity in psychiatric practice. Nursing Ethics, 4(6), 472–482. 10.1177/096973309700400604 9416106

[inr13066-bib-0028] Marshman, C. , Hansen, A. & Munro, I. (2022) Compassion fatigue in mental health nurses: a systematic review. Journal of Psychiatric and Mental Health Nursing, 29(4), 529–543. 10.1111/jpm.12812 34874593

[inr13066-bib-0029] Mersin, S. , İbrahimoğlu, Ö. , Çağlar, M. & Akyol, E. (2020) Compassionate love, burnout and professional commitment in nurses. Journal of Nursing Management, 28(1), 72–81. 10.1111/jonm.12892 31642139

[inr13066-bib-0030] Nas, E. & Sak , R. (2020) Compassion and compassion‐focused therapy. Manisa Celal Bayar University Journal of Social Sciences, 18(1), 64–84.

[inr13066-bib-0031] Oğuzhan, G. , Aydın, G.Z. & ve Bölükbaşı, F.B. (2019) Determination of nursing moral sensitivity: a state hospital example. Journal of Health Academics, 6(2), 91–99.

[inr13066-bib-0032] Ondrejková, N. & Halamová, J. (2022) Qualitative analysis of compassion fatigue and coping strategies among nurses. International Journal of Nursing Sciences, 9(4), 467–480. 10.1016/j.ijnss.2022.09.007 36285079 PMC9587405

[inr13066-bib-0033] Özcan, Z. (2020) The psychology of compassion (2nd edn.). Istanbul: Çamlıca Publications.

[inr13066-bib-0034] Özgünay, Ş.E. , Eminoğlu, Ş. , Önen, S. , Gürbüz, H. & Kılıçarslan, N. (2022) Compassion and chronic fatigue in anesthesiologists and intensive care unit workers in the COVID‐19 pandemic: a descriptive study. Turkiye Klinikleri Journal of Anesthesiology Reanimation, 20(3), 89–96. 10.5336/anesthe.2022-88989

[inr13066-bib-0035] Özhan, B. (2022) Compassion fatigue of intensive care nurses in the Covid‐19 pandemic, (master's thesis). İstanbul: İstanbul University, Institute of Health Sciences

[inr13066-bib-0036] Özkan, A. (2021). Determination of compassion and compassion fatigue in intensive care nurses, (Master's Thesis). Erzurum: Atatürk University, Institute of Health Sciences, Department of Internal Medicine Nursing.

[inr13066-bib-0037] Powell, S.K. (2020) Compassion fatigue. Professional Case Management, 25(2), 53–55. 10.1097/NCM.0000000000000418 32000203

[inr13066-bib-0038] Storm, J. & Chen, H.C. (2021) The relationships among alarm fatigue, compassion fatigue, burnout and compassion satisfaction in critical care and step‐down nurses. Journal of Clinical Nursing, 30(3–4), 443–453. 10.1111/jocn.15555 33174282

[inr13066-bib-0039] Tambag, H. , Mansuroğlu, S. & Yıldırım, G. (2018) Determination of the spiritual support perception of intensive care unit nurses: a pilot study. Journal of Contemporary Medicine, 8(2), 159–164.

[inr13066-bib-0040] Tanrıkulu, G. & Ceylan, B. (2021) Level of compassion and compassion fatigue in nurses working ın pediatric clinics. Journal of Health Sciences, 30(1), 31–36.

[inr13066-bib-0041] Tazegün, A & Çelebioğlu, A. (2016) Moral sensitivity levels of pediatric nurses and effective factors. Journal of İzmir Dr. Behçet Uz Pediatrics Hospital, 6(2), 97–102.

[inr13066-bib-0042] Temiz, Z. , Öztürk, D. , Ünver, S. , Tohumat, Ş.G. , Akyolcu, N. , Kanan, N. & ve Fethiye, N. (2017) Determining ethical sensitivity of nurses employed in surgical units. Journal of Anatolia Nursing and Health Sciences, 20(2), 83–89.

[inr13066-bib-0043] Tosun, H. (2018) Moral sensitivity questionnaire (MSQ): Turkish adaptation of the validity and reliability. Journal of Contemporary Medicine, 8(4), 316–321.

[inr13066-bib-0044] Uruf, S. (2022) Determining ethical sensitivity of nurses employed in surgical units, (Master's Thesis). Istanbul: Maltepe University, Institute of Health Sciences.

[inr13066-bib-0045] Ünlügedik, M. & Akbaş, E. (2023) The effect of spiritual well‐being on compassion fatigue among intensive care nurses: a descriptive study. Intensive and Critical Care Nursing, 77, 103432. 10.1016/j.iccn.2023.103432 37075662

[inr13066-bib-0046] Vafaeinia, R. , Baloochi Beydokhti, T. , Moghadam Abbaspour, L. & Ajamzibad, H. (2024) Spiritual health, nursing stress, compassion fatigue and COVID‐19: a descriptive correlational study. Journal of Research and Health, 14(1), 9–9.

[inr13066-bib-0047] Walker, W. (2020) Support after patient death in the intensive care unit: why ‘I’ is an important letter in grief. Nursing in Critical Care, 25, 266–268. 10.1111/nicc.12534 32815295

[inr13066-bib-0048] Willemse, S. , Smeets, W. , Van Leeuwen, E. , Nielen‐Rosier, T. , Janssen, L. & Foudraine, N. (2020) Spiritual care in the intensive care unit: an integrative literature research. Journal of Critical Care, 57, 55–78. 10.1016/j.jcrc.2020.01.026 32062288

[inr13066-bib-0049] Xie, W. , Chen, L. , Feng, F. , Okoli, C.T. , Tang, P. , Zeng, L. & Wang, J. (2021) The prevalence of compassion satisfaction and compassion fatigue among nurses: a systematic review and meta‐analysis. International Journal of Nursing Studies, 120, 103973. 10.1016/j.ijnurstu.2021.103973 34102372

[inr13066-bib-0050] Yılmaz, D. , Düzgün, F. , Yılmaz, D.U. , Korhan, E. A. & ve Dikmen, Y. (2018) Examination of ethical sensitivity and related factors of nurses in internal clinics: an example of university hospital. E‐journal of Dokuz Eylül University Nursing Faculty, 11(2), 157–163.

[inr13066-bib-0051] Yorulmaz, D.S. (2021) Investigation of the moral sensitivity and affecting factors of nurses. Turkiye Klinikleri Journal of Medical Ethics‐Law and History, 29(1), 86–93. 10.5336/mdethic.2020-73404

